# 2,6-Bis(1,4,7,10-tetraazacyclododecan-1-ylmethyl)pyridine and Its Benzene Analog as Nonmetallic Cleaving Agents of RNA Phosphodiester Linkages

**DOI:** 10.3390/ijms160817798

**Published:** 2015-08-03

**Authors:** Luigi Lain, Salla Lahdenpohja, Harri Lönnberg, Tuomas Lönnberg

**Affiliations:** Department of Chemistry, University of Turku, Vatselankatu 2, FIN-20014 Turku, Finland; E-Mails: luilai@utu.fi (L.L.); saorla@utu.fi (S.L.); harlon@utu.fi (H.L.)

**Keywords:** RNA, phosphodiester, cleavage, isomerization, catalysis, azacrown

## Abstract

2,6-Bis(1,4,7,10-tetraazacyclododecan-1-ylmethyl)pyridine (**11a**) and 1,3-bis(1,4,7,10-tetraazacyclododecan-1-ylmethyl)benzene (**11b**) have been shown to accelerate at 50 mmol·L^−1^ concentration both the cleavage and mutual isomerization of uridylyl-3′,5′-uridine and uridylyl-2′,5′-uridine by up to two orders of magnitude. The catalytically active ionic forms are the tri- (in the case of **11b**) tetra- and pentacations. The pyridine nitrogen is not critical for efficient catalysis, since the activity of **11b** is even slightly higher than that of **11a**. On the other hand, protonation of the pyridine nitrogen still makes **11a** approximately four times more efficient as a catalyst, but only for the cleavage reaction. Interestingly, the respective reactions of adenylyl-3′,5′-adenosine were not accelerated, suggesting that the catalysis is base moiety selective.

## 1. Introduction

**Figure 1:**
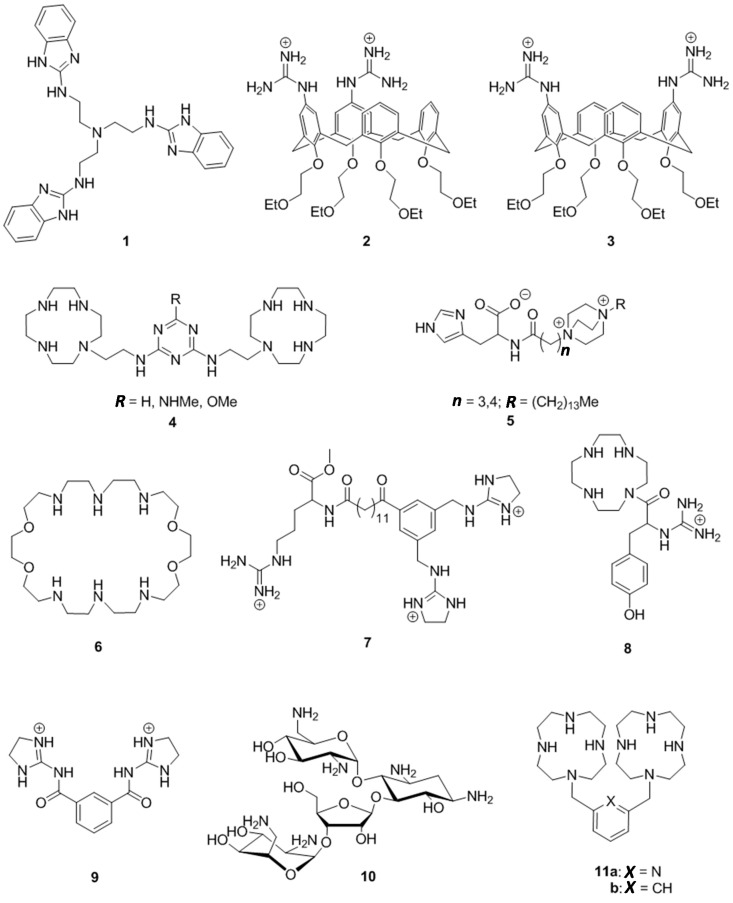
Representative examples of nonmetallic cleaving agents described in the literature.

Artificial ribonucleases, *i.e.*, small molecular compounds that catalyze the cleavage of RNA phosphodiester linkages under neutral conditions, have been the subject of continuous interest since the early 1990s [1,2,3,4]. The most active catalysts reported so far are complexes of transition metals and lanthanides, exhibiting half-lives of the order of 10 h for the cleavage of dinucleoside-3′,5′-monophosphates at 1.0 mmol·L^−1^ concentration of the complex at neutral pH and 25 °C [5,6]. Conjugation to an oligonucleotide or peptide nucleic acid (PNA) confers such agents sequence selectivity, turning them into real enzyme mimics [7]. Hybridization between the guiding oligonucleotide and the target sequence increases the local concentration and, hence, activity of the catalytic moiety. This interaction, when sufficiently strong, may render catalysis by the artificial ribonuclease essentially concentration-independent. For example, a PNA-conjugate of Cu^2+^: 2,9-dimethylphenanthroline cleaves a bulge in the target RNA opposite to the Cu^2+^ complex with a half-life as low as 30 min at 4 μmol·L^−1^ concentration of both the artificial RNase and the substrate, at pH 7.4 and 37 °C [8]. While metal ion based artificial RNases are undoubtedly useful for *in vitro* tailoring of large RNA molecules, a sufficient supply of metal ions under the metal-deficient intracellular conditions may become a rate-limiting factor. Purely organic cleaving agents (Figure 1) do not suffer from the same limitation, but the desired cleaving activity may be more difficult to achieve.

Several of the most active non-metallic cleaving agents known are derivatives of guanidine. Tris[2-(benzimidazol-2-yl)ethyl]amine (**1**) at 1 mmol·L^−^^1^ concentration cleaves RNA at pH 6.0, the half-life for the fission of a single RNA phosphodiester linkage being 60 h at 37 °C [9]. The cleaving agent tends to aggregate under these conditions, and the efficiency of **1** in its non-aggregated form is somewhat lower [10]. Diguanidinocalix[4]arenes (**2** and **3**) cleave dinucleoside-3′,5′-monophosphates at a comparable rate in 80% aq. DMSO at pH 10.4 [11]. The half-lives range from 3 to 300 h at 50 °C, depending on the identity of the base moieties. Melamine constructs anchored via Zn^2+^:cyclen moieties to uracil bases (**4**) accelerate the cleavage of UpU by two orders of magnitude at neutral pH (*t*_½_ = 12 h at 90 °C) [12,13]. The other markedly efficient non-metallic agents include quaternized 1,4-diazabicyclo[2,2,2]octane conjugates of histidine (**5**). They exhibit at 0.5 mmol·L^−^^1^ concentration half-lives of approximately 40 h for the cleavage of a single phosphodiester bond within a 10-mer oligoribonucleotide at pH 7.0 and 37 °C [14]. Among polyamines, the tetracation of 1,4,16,19-tetraoxa-7,10,13,22,25,28-hexaazacyclotriacontane (**6**) is catalytically exceptionally active (at [**6**] = 5 mmol·L^−^^1^, *t*_½_ = 200 h at 50 °C) [15]. In addition, several non-metallic agents cleave selectively some particular phosphodiester bond within large RNA molecules, although the catalytic activity towards short oligonucleotides is low. The trans-activation response (TAR) element of HIV RNA, for example, is cleaved by guanidinium based structures **7** [16], **8** [17] and **9** [18] and neomycin B (**10**) [19]. Multifunctional constructs that contain amino, amido and imidazolyl groups [20,21,22,23,24] or incorporate a 1,4-diazabicyclo[2.2.2]octane dimer attached to a hydrophobic tail [25,26] have, in turn, been shown to cleave tRNA molecules at specific sites, usually at 5′-CpA-3′ sites within single-stranded regions. We now report on 2,6-bis(1,4,7,10-tetraazacyclododecan-1-ylmethyl)pyridine (**11a**) and 1,3-bis(1,4,7,10-tetraazacyclododecan-1-ylmethyl)benzene (**11b**) as non-metallic cleaving agents. Both **11a** and **11b** feature two cyclen rings connected by a relatively rigid *m*-xylylene-type linker for a high density of amino groups. Somewhat unexpectedly, **11a**,**b** seem to exhibit a selectivity for uracil bases, normally associated with Zn^2+^ chelates of cyclen. The cleaving activity of uncomplexed **11a**,**b**, however, is considerably lower than what is typically observed with related Zn^2+^ complexes.

## 2. Results

### 2.1. Synthesis of Cleaving Agents **11a** and **11b**

2,6-Bis(1,4,7,10-tetraazacyclododecan-1-ylmethyl)pyridine (**11a**) and 1,3-bis(1,4,7,10-tetraazacyclododecan-1-ylmethyl)benzene (**11b**) were obtained by the reaction of 1,4,7-tris(*tert*-butoxycarbonyl)-1,4,7,10-tetraazacyclododecane with 2,6-bis(bromomethyl)pyridine and 1,3-bis(bromomethyl)benzene, respectively, in MeCN in the presence of K_2_CO_3_, followed by removal of the *tert*-butoxycarbonyl protections with TFA in a mixture of MeOH and CH_2_Cl_2_ [27].

### 2.2. Determination of the pK_a_ Values of the Cleaving Agents

**Figure 2:**
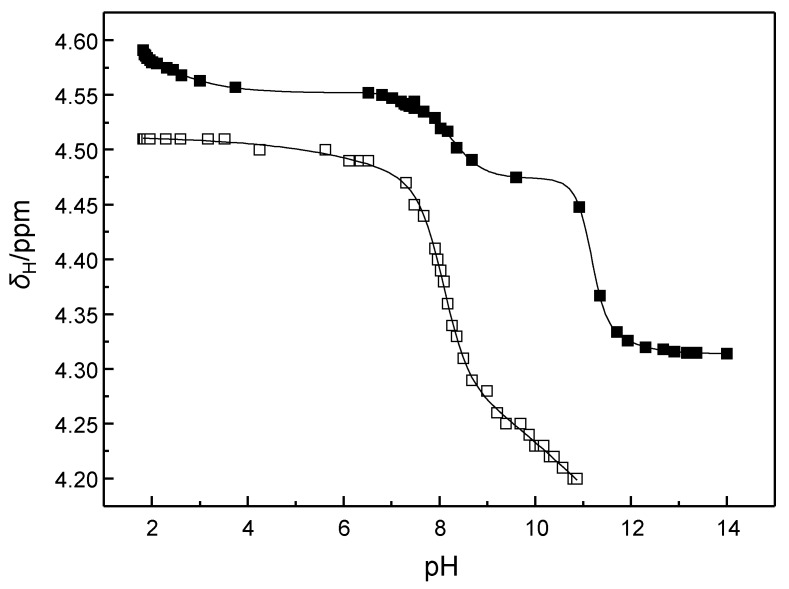
Chemical shift of the methylene protons of **11a** (■) and **11b** (□) as a function of pH; *T* = 90 °C; [phosphoric acid] = 0.10 mol·L^−1^. The lines have been added to illustrate the trends of chemical shift *versus* pH.

The p*K*_a_ values of **11a** and **11b** have been previously determined by potentiometric titration at 25 °C (*I* = 0.1 mol·L^−1^) [28]. Both of the compounds undergo five consecutive protonations on increasing the acid concentration to 0.1 mol·L^−1^. The p*K*_a_ values obtained with **11a** are 11.08, 10.14, 8.97, 7.84 and 2.30. Interestingly, replacement of the pyridine nitrogen atom with methine group has only a minor effect on the protolytic equilibria, the p*K*_a_ values of **11b** being 11.25, 10.02, 8.90, 7.97 and 2.07. To obtain the values at 90 °C, *i.e.*, under the conditions of kinetic experiments, ^1^H NMR spectrometric titrations in D_2_O were carried out. The compound (**11a** or **11b**) was dissolved in phosphoric acid (0.10 mol·L^−1^) and 1.0 mol·L^−1^ sodium deuteroxide in D_2_O was gradually added. A downfield shift of the ^31^P signal of the buffer and an upfield shift of the methylene signals of **11a** and **11b** were observed (Figure 2). The p*K*_a_ values were then calculated on the basis of the p*K*_a_ values of phosphoric acid at 90 °C [29]. The values obtained with **11a** were 11.34 ± 0.22, 11.18 ± 0.11, 8.26 ± 0.07, 6.67 ± 0.14 and 2.60 ± 0.14. As the third p*K*_a_ value of phosphoric acid was extrapolated based on values obtained at much lower temperatures, the two most alkaline p*K*_a_ values of **11a** are subject to some uncertainty. This uncertainty did not affect our results, however, since only the p*K*_a_ values of the tri- (SH_3_^3+^), tetra- (SH_4_^4+^) and pentaprotonated (SH_5_^5+^) species, 8.26, 6.67 and 2.60, respectively were needed to analyze the kinetic data. For the same reason, titration of **11b** was not extended beyond pH 11.0. The p*K*_a_ values of the tri- (SH_3_^3+^) and tetraprotonated (SH_4_^4+^) species of **11b** were 8.4 ± 0.2 and 7.5 ± 0.6, respectively, p*K*_a_ of the pentaprotonated species being too low to be determined by this method. It is worth noting that the p*K*_a_ values were obtained in a 0.1 mol·L^−1^ phosphate buffer, while the kinetic measurements were carried out in 1.0 mol·L^−1^ NaClO_4_. Owing to this difference in background electrolyte, the p*K*_a_ values under the conditions of kinetic measurements may slightly deviate from those determined by NMR titration. Most likely, none of the cyclen nitrogens is exceptionally basic, but the proton distribution is statistic.

### 2.3. Kinetic Measurements

The cleavage of uridylyl-3′,5′-uridine (3′,5′-UpU) to uridine 2′,3′-cyclic phosphate and uridine, and its concurrent isomerization to 2′,5′-UpU was studied in excess of cleaving agent (**11a** or **11b**) under acidic conditions where **11a** and **11b** exist in tri-, tetra- or pentacationic forms. The measurements were carried out at different concentrations of **11a** or **11b** in the range from 10 to 70 mmol·L^−1^. At each concentration, the proportions of ionic forms were altered by titration with 3.5, 3.8, 4.5 or 5.0 equivalents (eq.) of perchloric acid. Under the most acidic conditions studied, concentration of free hydronium ion became significant and was taken into account when calculating the pH of the reaction solutions. For the same reason, the buffer ratios were not entirely constant over all the buffer concentrations employed. These deviations, however, were deemed small enough to not significantly affect the results of the kinetic measurements. Sodium perchlorate (1.0 mol·L^−1^) was used as a background electrolyte. The starting concentration of UpU was 0.50 mmol·L^−1^.

**Figure 3:**
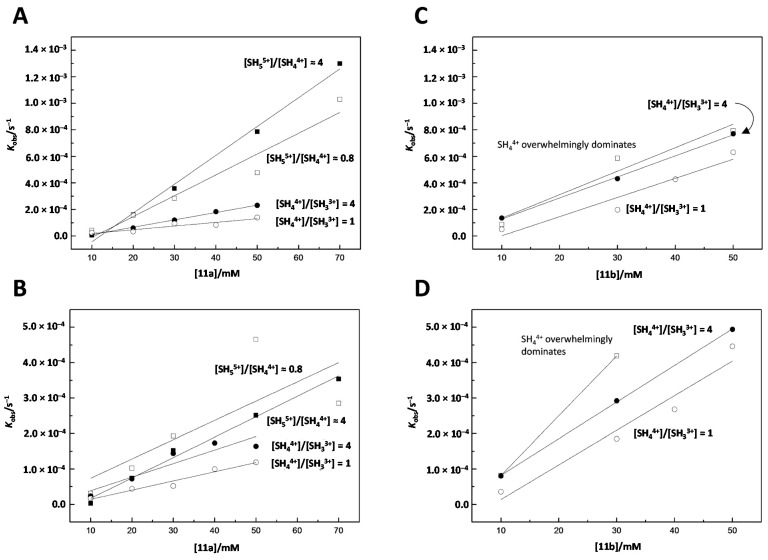
Observed pseudo first-order rate constants for (**A**) cleavage and (**B**) isomerization of UpU as a function of [**11a**] and (**C**) cleavage and (**D**) isomerization of UpU as a function of [**11b**]; *T* = 90 °C, *I*(NaClO_4_) = 1.0 mol·L^−1^. The catalyst (**11a** or **11b**) was titrated alternatively with 3.5 (○), 3.8 (●), 4.5 (□) or 5.0 (■) eq. of perchloric acid.

The pseudo first-order rate constants obtained for the cleavage and isomerization reactions as functions of [**11a**] and [**11b**] are presented in Figure 3. As seen, both reactions exhibit a first-order dependence on the catalyst concentration with both **11a** and **11b** and regardless of the distribution among the various ionic forms (Equation (1)).

(1) $$k_{\text{obs}}=k_{\text{cat}}{\left[\text{catalyst}\right]}_{\text{total}}+k_{\text{uncat}}$$

where *k*_cat_ and *k*_uncat_ refer to the second-order rate constant for the catalyzed reaction and the first-order rate constant for the uncatalyzed (background) reaction, respectively. In most cases, the catalyzed reaction was overwhelmingly faster than the uncatalyzed one, precluding reliable determination of the latter term.

The catalytic activity of **11a** is increased with the increasing extent of protonation. Curiously, with **11b** this dependence is much weaker. In solutions where the catalyst has been titrated with 3.5 eq. of perchloric acid, [SH_4_^4+^] = [SH_3_^3+^] and, hence, the pH is 6.7 and 7.5 for **11a** and **11b** respectively. Under these conditions, the first-order rate constants for the cleavage of UpU in the absence of any cleaving agents are approximately 3 × 10^−7^ and 2 × 10^−6^ s^−1^, respectively [30]. Uncatalyzed isomerization is pH-independent with a first-order rate constant of 10^−6^ s^−1^, the conversion to both directions taking place as readily. Accordingly, accelerations of more than two orders of magnitude are achieved with both **11a** and **11b** at 50 mmol·L^−1^ concentration. Catalysis by **11a** was also studied with adenylyl-3′,5′-adenosine (3′,5′-ApA) as the substrate. Virtually no rate acceleration could be observed, suggesting that the reasonably high cleaving activity of **11a** depends on the presence of uracil base. It is also worth noting that monomeric 1,4,7,10-tetraazacyclododecane (cyclen) did not catalyze the cleavage or isomerization of 3′,5′-UpU.

**Figure 4:**
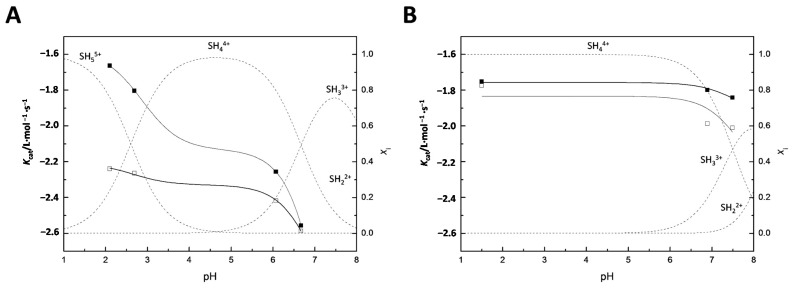
pH-rate profiles for the (**A**) **11a**- and (**B**) **11b**-catalyzed cleavage (■) and isomerization (□) of UpU; *T* = 90 °C, *I*(NaClO_4_) = 1.0 mol·L^−1^.

Figure 4 shows the pH-rate profiles for the **11a**- and **11b**-catalyzed cleavage and isomerization of UpU. For reference, speciation curves for the various ionic forms of the catalysts have been included as well. Again, the pH-dependence of the catalytic activity of **11a** and the relative pH-independence of the catalytic activity of **11b** is evident. In the pH range of the kinetic experiments, protolytic equilibria of the catalysts are adequately described in terms [SH_3_]^3+^, [SH_4_]^4+^ and (in the case of **11a**) [SH_5_]^5+^. The observed second-order rate constants for **11a** and **11b** may, hence, be expressed by Equations (2) and (3), respectively.

(2) $$k_{\text{cat}}\left(11a\right)=\frac{k_{5}{\left[{\text{H}}^{+}\right]}^{2}+k_{4}K_{\text{a}5}\left[{\text{H}}^{+}\right]+k_{3}K_{\text{a}4}K_{\text{a}5}}{{\left[{\text{H}}^{+}\right]}^{2}+K_{\text{a}5}\left[{\text{H}}^{+}\right]+K_{\text{a}4}K_{\text{a}5}}$$

(3) $$k_{\text{cat}}\left(11b\right)=\frac{k_{4}\left[{\text{H}}^{+}\right]+k_{3}K_{\text{a}4}}{\left[{\text{H}}^{+}\right]+K_{\text{a}4}}$$

where *k*_3_, *k*_4_ and *k*_5_ are the second-order rate constants for the reactions catalyzed by the tri-, tetra- and pentaprotonated species, respectively. *K*_a4_ and *K*_a5_ are the acid dissociation constants for the tetra- and pentaprotonated species, respectively. The acid dissociation constants were obtained by NMR spectrometric titrations as described above and the rate constants by non-linear least-squares fitting of the observed second-order rate constants (*k*_cat_) to Equations (2) and (3). The values are summarized in Table 1.

**Table 1:** Second-order rate constants for the cleavage and isomerization of UpU, catalyzed by the tri-, tetra- and pentacationic forms of **11a** and **11b**; *T* = 90 °C, *I*(NaClO_4_) = 1.0 mol·L^−1^.

Rate Constant	11a		11b	
	Cleavage	Isomerization	Cleavage	Isomerization
*k*_5_/10^−3^ L·mol^−1^·s^−1^	26.1 ± 0.2	6.2 ± 0.2	N/A *^a^*	N/A *^a^*
*k*_4_/10^−3^ L·mol^−1^·s^−1^	7.37 ± 0.06	4.7 ± 0.1	17.5 ± 0.4	15 ± 3
*k*_3_/10^−3^ L·mol^−1^·s^−1^	N/A *^b^*	0.5 ± 0.2	11.1 ± 0.8	4 ± 6

*^a^* The concentration of the pentacationic form of **11b** is negligible under the experimental conditions; *^b^* Fitting to Equation (2) gave a negative value.

Comparison of the second order rate constants of the cleavage reaction catalyzed by **11a** suggests that SH_5_^5+^ is approximately 4 times as active as SH_4_^4+^ and the latter is, in turn, at least one order of magnitude more active than SH_3_^3+^. In fact, whether the tricationic form of **11a** contributes at all to the catalysis is questionable. Unlike cleavage, isomerization is catalyzed approximately equally by the penta- and tetracationic forms of **11a**. Even the tricationic species appears to exhibit some activity, although the reliability of this result is debatable. The benzene analog **11b** does not undergo a fifth protonation under conditions of the kinetic runs. Catalysis of cleavage and isomerization by SH_4_^4+^ predominates over a wide pH range and is approximately three times as efficient with **11b** as with **11a**. In the case of **11b**, catalytic activity of SH_3_^3+^ is also evident although lower than that of SH_4_^4+^.

## 3. Discussion

### 3.1. Base-Selectivity of **11a**

The uracil-selectivity of **11a** parallels the behavior previously observed with the dinuclear Zn^2+^ complex of a structural analog, 1,3-bis(1,5,9-triazacyclododecan-3-yloxymethyl)benzene. In that case, one of the Zn^2+^(azacrown) moieties anchors the cleaving agent to a uracil base, the other moiety cleaving the neighboring phosphodiester linkage. Accordingly, 3′,5′-ApU and 3′,5′-UpA are cleaved much faster than 3′,5′-ApA or other dinucleoside monophosphates that do not contain uracil bases. 3′,5′-UpU is not cleaved, suggesting that both Zn^2+^(azacrown) moieties are engaged in uracil binding, but the catalytic activity is restored by addition of a third azacrown ligand [31,32,33]. A key difference between the present results and those obtained in the presence of Zn^2+^ is, however, seen in the respective pH-rate profiles. In the latter case, efficient catalysis was only observed under neutral and mildly alkaline conditions (where coordination of Zn^2+^ to deprotonated N3 atom of uracil prevails) whereas in the present case, catalysis by **11a** and **11b** was most pronounced under acidic conditions. In fact, under such conditions, Zn^2+^ would not even be appreciably coordinated by cyclen [12].

**Figure 5:**
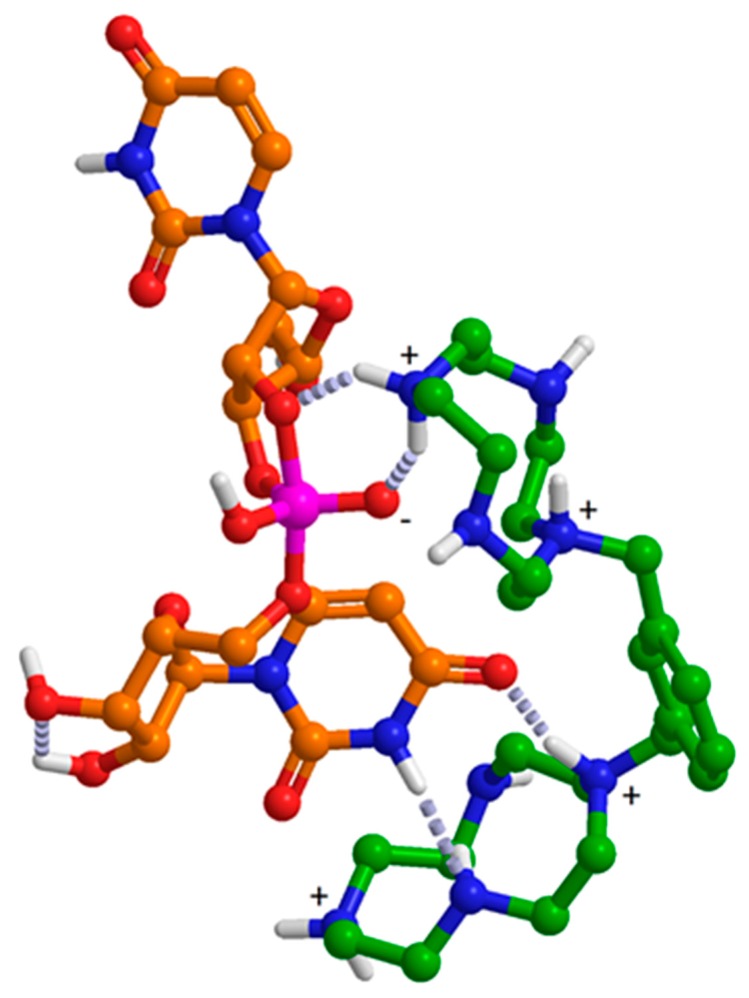
MOPAC (PM6)-minimized structure for tetracationic **11b** (green) hydrogen bonded (dashed lines) to the monoanionic, pentacoordinate intermediate (orange) of UpU cleavage and hydrolysis.

Compared to their metal chelates, binding of uncomplexed azacrowns to nucleobases is much weaker and has been studied much less extensively. One could, however, envision a binding mode unique to uracil (and thymine) bases. The two oxo substituents may both accept a hydrogen bond from a single dicationic cyclen molecule. In adenine, orientation of the two potential hydrogen bond acceptors (N1 and N7) is not conducive for such binding. In the case of tetracationic **11a** (or **11b**) and UpU, one of the cyclen moieties would bind electrostatically to the anionic phosphodiester linkage and the other one via hydrogen bonding to one of the uracil bases (Figure 5). While the former interaction is undoubtedly stronger, the inefficiency of cyclen itself as a catalyst indicates that the latter is also vital in achieving a sufficient local concentration. Similar behavior has also been reported with other bifunctional cleaving agents, notably the calixarene derivatives **2** and **3** that cleave 3′,5′-GpU, 3′,5′-GpG and 3′,5′-UpU from one to two orders of magnitude faster than the other dinucleoside monophosphates [11]. Attempts to quantify the affinity of cyclen to uridine and adenosine by NMR titrations were unsuccessful-significant binding was only observed under conditions where the uracil base is deprotonated (data presented in Figure S1 in the supporting information). Apparently interactions between **11a** (or **11b**) and dinucleoside monophosphates are too complex to be studied by this simplified model system.

### 3.2. Mechanism of the Catalysis by **11a** and **11b**

In striking contrast to the Zn^2+^(azacrown)-based catalysts, **11a** and **11b** catalyze isomerization almost as efficiently as cleavage. To the best of our knowledge, the present study is the first report on catalysis of isomerization of RNA phosphodiester linkages by a non-metallic small molecular entity. The tetracationic forms of **11a** and **11b** facilitate both cleavage and isomerization to approximately the same extent, making stabilization of the common phosphorane intermediate an attractive mechanistic alternative. This together with the observed base-selectivity suggests that **11a** and **11b** interact with 3′,5′-UpU by simultaneous binding of the doubly protonated cyclen ligands to the uracil base and the internucleosidic phosphodiester linkage. Evidently, binding is enforced upon formation of the phosphorane intermediate by attack of the 2′-OH. Protonation of the pyridine nitrogen makes **11a** a somewhat better catalyst, but only in the case of cleavage. The additional acceleration could be tentatively attributed to assistance of the departure of the 5′-oxygen by hydrogen bonding from the protonated pyridine nitrogen.

## 4. Experimental Section

### 4.1. General Methods

NMR spectra were recorded on a Bruker Avance 400, 500, or 600 NMR spectrometer and the mass spectra on a Bruker Daltonics Micro-TOF-Q high resolution mass spectrometer. The ^1^H NMR chemical shifts were referenced to internal TMS. The reagents, including 1,4,7-tris(*tert*-butoxycarbonyl)-1,4,7,10-tetraazacyclododecane, were commercial products that were used as received.

### 4.2. 2,6-Bis(1,4,7,10-tetraazacyclododecan-1-ylmethyl)pyridine (**11a**)

Compound **11a** was prepared as described previously by Xiang *et al* [27]*.* Accordingly, 2,6-bis(bromomethyl)pyridine was treated with 1,4,7-tris(*tert*-butoxycarbonyl)-1,4,7,10-tetraazacyclododecane in MeCN in the presence of K_2_CO_3_ and the *tert*-butoxycarbonyl protections were removed with TFA in 1:4 (*v*/*v*) mixture of MeOH and CH_2_Cl_2_. The product was converted to free base with strong anion-exchange resin (Dowex 1X2, mesh 200, OH^−^-form) in water. The ^1^H NMR spectrum of the material thus obtained was identical to that previously reported for **11a**. ^1^H NMR (D_2_O, 500 MHz): δ 7.72 (t, *J* = 7.8 Hz, 1H), 7.35 (d, *J* = 7.8 Hz, 2H), 3.64 (s, 4H), 2.50–2.68 (m, 32H). ^13^C NMR (D_2_O, 125.8 MHz): δ 158.4, 138.3, 122.9, 59.6, 50.9, 45.0, 44.2, 43.2. HRMS (ESI^+^): *m*/*z* calcd 448.3876 obsd 448.3860 [M + H]^+^.

### 4.3. 1,3-Bis(1,4,7,10-tetraazacyclododecan-1-ylmethyl)benzene (**11b**)

Compound **11b** was prepared as described above for **11a** except that 1,3-bis(bromomethyl)benzene was used instead of 2,6-bis(bromomethyl)pyridine as the starting material. The ^1^H NMR spectrum of the product was identical to that previously reported for **11b**. ^1^H NMR (D_2_O, 500 MHz): δ 7.32 (t, *J* = 8.0 Hz, 1H), 7.23 (s, 1H), 7.16 (d, *J* = 8.0 Hz, 2H), 3.58 (s, 4H), 2.55–2.78 (m, 32H). ^13^C NMR (D_2_O, 125.8 MHz): δ 138.4, 130.7, 129.0, 128.6, 57.5, 50.6, 44.8, 43.3, 43.2. *m*/*z* calcd 447.3918 obsd 447.3913 [M + H]^+^.

### 4.4. Determination of the pK_a_ Values

The p*K*_a_ values of **11a** and **11b** were determined by NMR spectrometric titrations at 90 °C in D_2_O buffered with 0.10 mol·L^−1^ phosphoric acid. Accordingly, compound **11a** or **11b** was dissolved in phosphoric acid (0.10 mol·L^−1^) and 1.0 mol·L^−1^ sodium deuteroxide in D_2_O was gradually added. The p*K*_a_ values were determined as inflection points of the plots of the ^1^H chemical shifts of the methylene protons as a function of pH. The pH axis, in turn, was calibrated on the basis of the observed ^31^P chemical shift and known p*K*_a_ values of phosphoric acid at 90 °C [29].

### 4.5. Kinetic Measurements

The reactions were carried out in sealed tubes immersed in a thermostated water bath (90.0 ± 0.1 °C). The pH of the reaction solutions were adjusted by titrating **11a** or **11b** (10–70 mmol·L^−1^) with 3.5, 3.8, 4.5 or 5.0 eq. of perchloric acid. In other words, **11a** and **11b** served as both cleaving agents and buffers. Sodium perchlorate (1.0 mol·L^−1^) was used as a background electrolyte. The initial concentration of 3′,5′-UpU and 3′,5′-ApA was 500 μmol·L^−1^. 4-Nitrophenol (1.0 mmol·L^−1^) was used as an internal standard. To rule out the possibility of nucleophilic catalysis by the phenolic internal standard, some of the kinetic runs were also carried out in the absence of this standard, with identical results.

The composition of the samples withdrawn at appropriate time intervals was analyzed by HPLC on a Hypersil-Keystone Aquasil C18 column (4 × 150 mm, 5 μm) using a mixture of MeCN and a 50 mmol·L^−1^ TRIS buffer (pH 8.1) containing 50 mmol·L^−1^ ammonium chloride as an eluent. The cleaving agents **11a** and **11b** were present in at least 20-fold excess in all samples and they eluted as broad peaks. To reduce the concentration of **11a** and **11b** to a level where these peaks would not overlap with those of the other components, the samples were diluted with water (1:10) before injection. For the first 8 min, isocratic elution with 100% buffer was applied, followed by a linear gradient from 0% to 15% of MeCN over 11 min. A constant flow rate of 1.0 mL·min^−1^ and a detection wavelength of 260 nm were employed. The observed retention times (*t*_R_, min) for 3′,5′-UpU, its reaction products and the internal standard were: 4.7 min (uridine), 10.8 min (4-nitrophenol), 14.7 min (2′,5′-UpU) and 15.7 (3′,5′-UpU). The products were characterized by spiking with authentic samples.

The molar absorptivities of the 2′- and 3′-isomers of UpU were assumed to be identical. Calculation of the rate constants for the cleavage and isomerization reactions was based on the signals of 3′,5′-UpU, 2′,5′-UpU and uridine. The signals of uridine monophosphates were not used as their integration was not entirely reproducible. The conversion of uridine monophosphates to uridine was, under the conditions employed, so slow that it did not appreciably affect the time-dependent concentration of uridine. In other words, uridine could be assumed to form exclusively by cleavage of UpU. Cleavage and isomerization of UpU obeyed the first-order rate law throughout the kinetic runs (a representative time profile is presented in Figure S2 in the supporting information), as could be expected given the large excess of the cleaving agents **11a** and **11b**. Pseudo-first-order rate constants for the cleavage and isomerization reactions were calculated by numerical fitting of the experimental data to the differential rate equations, (data summarized in Tables S1–S4 in the supporting information).

## 5. Conclusions

The bis-cyclen cleaving agents **11a** and **11b** catalyze the cleavage and isomerization of UpU under neutral and acidic conditions, in all likelihood by lowering the negative charge on the phosphodiester linkage through interaction with a doubly protonated cyclen moiety. Additional binding of the other cyclen moiety to one of the uracil bases is also needed for efficient catalysis. Compared to the other non-metallic cleaving agents published so far, the catalytic activity of **11a** and **11b** appears to be somewhat lower than that of the most active ones (**1** and **5**). Quantitative comparison is difficult, since the experimental data for various catalysts refer to very different conditions. The highest cleaving activities have been observed with **1** and **5** by using oligonucleotide targets [9,14]. With dinucleoside monophosphates, the most efficient cleaving agents are the calixarene derivatives **2** and **3**, but in that case the data was obtained in 80% aqueous DMSO [11].

## Supplementary Materials

Supplementary materials can be found at http://www.mdpi.com/1422-0067/16/08/17798/s1.
