# Supplementary material for: 2,6-Bis(1,4,7,10-tetraazacyclododecan-1-ylmethyl)pyridine and Its Benzene Analog as Nonmetallic Cleaving Agents of RNA Phosphodiester Linkages
Source: Int J Mol Sci. 2015 Aug 3;16(8):17798–811. doi: 10.3390/ijms160817798 (PMC4581222; doi:10.3390/ijms160817798)
Supplement: Supplementary file 1 [file ijms-16-17798-s001.pdf]

Supplementary Information

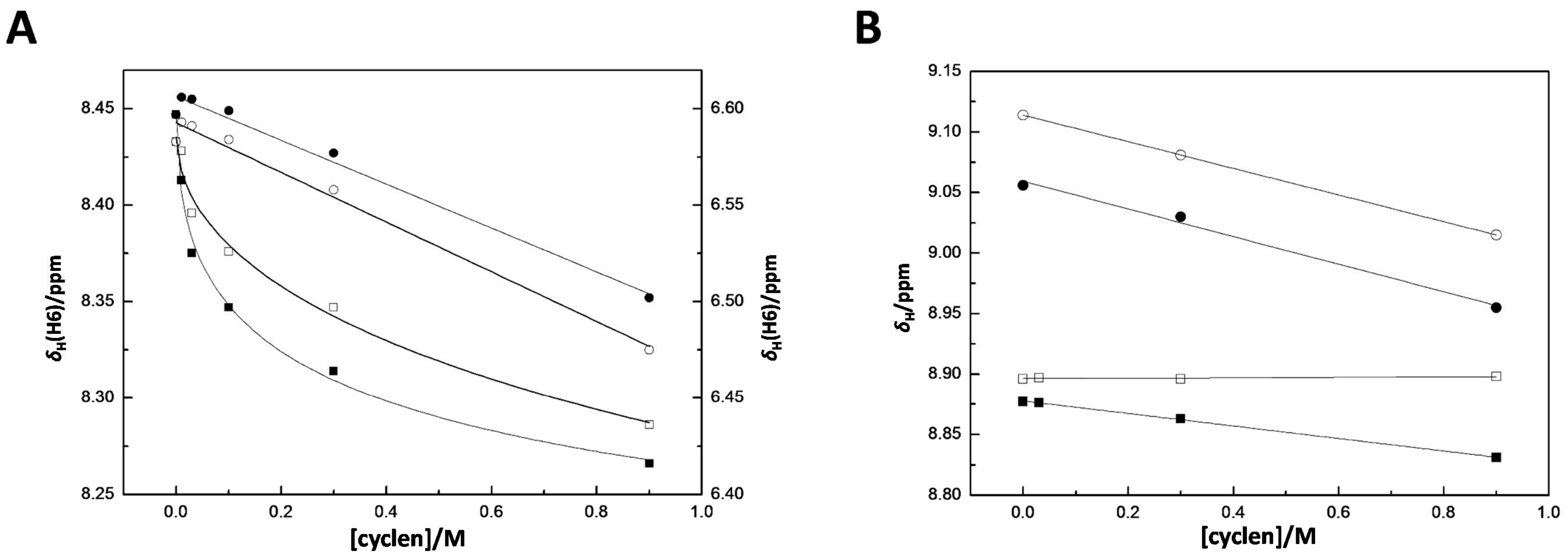

Figure S1. Dependence of the chemical shift of (**A**) H5 (hollow symbols) and H6 (solid symbols) of uridine and (**B**) H2 (hollow symbols) and H8 (solid symbols) of adenosine on the concentration of cyclen in D_2O at 90 °C. Cyclen was protonated with either 1.2 (squares) or 2.1 (circles) eq. of deuterium chloride prior to the NMR titration.

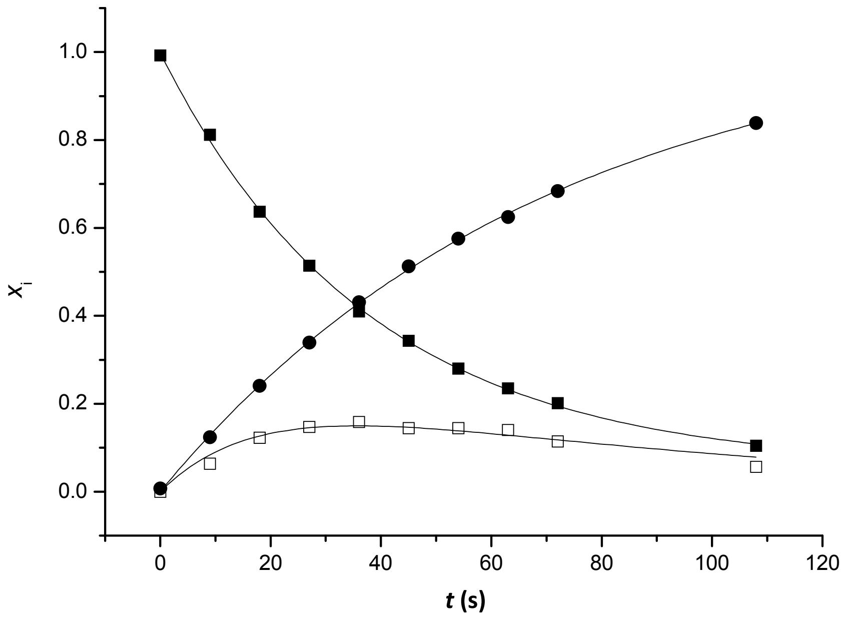

Figure S2. Time-dependent mole fractions of 3′,5′-UpU (■), 2′,5′-UpU (□) and uridine (●) in 20 mM **11a** titrated with 4.5 equivalents (eq.) of $HClO_4$; T = 90 °C, pH = 2.6, I ($NaClO_4$) = 1.0 mol·L^{-1}.

Table S1. Observed pseudo first-order rate constants for the cleavage of UpU in the presence of **11a**; T = 90 °C, I($NaClO_4$) = 1.0 mol·L^{-1}.

[11a]/mmol·L^{-1}	k_{obs}(cleavage)/10^{-5}·s^{-1}			
	+ 3.5 eq. $HClO_4$	+ 3.8 eq. $HClO_4$	+ 4.5 eq. $HClO_4$	+ 5.0 eq. $HClO_4$
10	2.44	1.51	4.12	0.48
20	3.44	6.01	15.7	15.9
30	9.41	11.9	28.4	35.7
40	8.28	18.3	-	-
50	13.9	23.1	47.7	78.6
70	-	-	103	130

Table S2. Observed pseudo first-order rate constants for the isomerization of UpU in the presence of **11a**; T = 90 °C, I($NaClO_4$) = 1.0 mol·L^{-1}.

[11a]/mmol·L^{-1}	k_{obs}(isomerization)/10^{-5}·s^{-1}			
	+ 3.5 eq. $HClO_4$	+ 3.8 eq. $HClO_4$	+ 4.5 eq. $HClO_4$	+ 5.0 eq. $HClO_4$
10	1.71	2.34	3.10	0.31
20	4.43	7.19	10.2	7.43
30	5.17	14.4	19.3	15.2
40	9.95	17.3	-	-
50	11.9	16.4	46.6	25.1
70	-	-	28.5	35.4

Table S3. Observed pseudo first-order rate constants for the cleavage of UpU in the presence of **11b**; $T = 90$ °C, $I(NaClO_4) = 1.0$ mol·L^{-1}.

[11b]/mmol·L^{-1}	k_{obs}(cleavage)/10^{-5}·s^{-1}		
	+ 3.5 eq. $HClO_4$	+ 3.8 eq. $HClO_4$	+ 4.5 eq. $HClO_4$
10	4.96	13.5	8.59
30	19.8	43.1	59
40	42.7	-	-
50	63.1	77	79

Table S4. Observed pseudo first-order rate constants for the isomerization of UpU in the presence of **11b**; $T = 90$ °C, $I(NaClO_4) = 1.0$ mol·L^{-1}.

[11b]/mmol·L^{-1}	k_{obs}(isomerization)/10^{-5}·s^{-1}		
	+ 3.5 eq. $HClO_4$	+ 3.8 eq. $HClO_4$	+ 4.5 eq. $HClO_4$
10	3.57	8.05	8.16
30	18.5	29.2	41.9
40	26.8	-	-
50	44.6	49.4	240
